# Low-temperature fluoride-assisted synthesis of mullite whiskers†

**DOI:** 10.1039/d0ra05997h

**Published:** 2020-08-24

**Authors:** Amanmyrat Abdullayev, Fabian Zemke, Aleksander Gurlo, Maged F. Bekheet

**Affiliations:** Fachgebiet Keramische Werkstoffe/Chair of Advanced Ceramic Materials, Institute of Materials Science and Technology, Technische Universität Berlin 10623 Berlin Germany maged.bekheet@ceramics.tu-berlin.de

## Abstract

Mullite is a promising material for advanced ceramic applications. The synthesis of mullite from oxides requires very high temperatures (*T* > 1000 °C). Here highly crystalline mullite whiskers with an average length and diameter of 2.37 ± 1.7 μm and 0.18 ± 0.11 μm, respectively, were synthesized by a fluoride-assisted method from aluminium sulfate, aluminium fluoride and fumed silica at a temperature as low as 800 °C.

## Abstract 2

Fluoride-assisted method for obtaining highly crystalline mullite whiskers at low temperature (∼800 °C) is reported.

## Graphical abstract

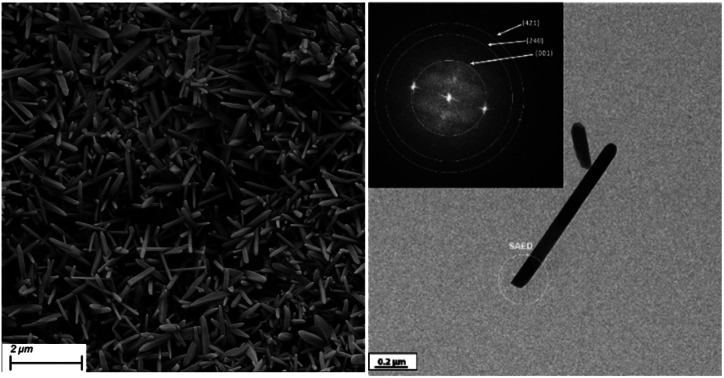

## Introduction

Mullite is an aluminosilicate with stoichiometries ranging from relatively silica-rich 3Al_2_O_3_·2SiO_2_ (3 : 2 mullite) to alumina-rich 2Al_2_O_3_·SiO_2_ (2 : 1 mullite). Superior properties, such as excellent creep resistance, high-temperature stability, good chemical stability, low thermal conductivity and low thermal expansion, made mullite's widespread utilization from conventional ceramics to advanced structural and functional ceramics.^1^ In the last few years, the synthesis of mullite has received more attention due to the low availability of mullite in natural rocks. Although various synthesis methods/approaches of mullite are reported, such as solid-state synthesis,^2^ sol–gel route,^3^ and the optical floating zone technique,^4^ most of them require very high temperatures to obtain well-crystalline mullite. Previous works showed that the mullite synthesis temperature depends on several factors, such as the homogeneity and chemical compositions of precursors, additives, impurities, and reaction atmosphere.^5^ For instance, crystalline mullite starts to appear from 980 °C onwards by calcination of ultra homogeneous sol–gel derived mullite precursors,^6^ while much higher temperatures (>1400 °C) are used to obtain mullite from mechanically mixed alumina and silica.^2^ Another approach commonly applied to reduce the synthesis temperature of mullite is to use fluorides (such as AlF_3_) as reactants or additives as well as to conduct synthesis in a fluor-containing atmosphere. This not only reduces the synthesis temperature of mullite but also facilitates the crystallization of mullite whiskers/materials with acicular crystal habit.^7^

According to Okada and Ōtsuka, the formation mechanisms of mullite in the presence of AlF_3_ can be described as follows:^8^

1 6AlF_3(s)_ + 3O_2(g)_ → 6AlOF_(g)_ + 12F_(g)_

2 Al_2_O_3(s)_ + 2F_(g)_ → 2AlOF_(g)_ + 0.5O_2(g)_

3 2SiO_2(s)_ + 8F_(g)_ → 2SiF_4(g)_ + 2O_2(g)_

4 6AlOF_(g)_ + 2SiF_4(g)_ + 3.5O_2(g)_ → 3Al_2_O_3_·2SiO_2(s)_ + 14F_(g)_

Although the formed intermediates fluorides AlOF and SiF_4_ have high vapour pressure at high temperatures and will promote the nucleation of mullite at about 900 °C, the formation of mullite is completed only at higher temperatures (≥1100 °C).^7,9–15^ Moreover, it is reported that the application of airflow over fluoride-based reactants or using compacted topaz can enhance mullite whisker growth with a length up to 250 μm at *T* ≥ 1200 °C due to adequate pressure of water/fluoride vapours.^9,10^ Recently, Rashad *et al.* reported that water vapours originated from aluminium fluoride trihydrate (AlF_3_·3H_2_O) during the synthesis promotes the nucleation of mullite at about 700 °C, but temperatures as high as 1200 °C were required to obtain highly crystalline mullite.^12,16,17^ In the presence of water vapours, the mullite could be formed through the reaction between gaseous AlF_3_, AlOF and SiF_4_ as follows:^18^

5 AlF_3(g)_ + 5AlOF_(g)_ + 2SiF_4(g)_ + 8H_2_O_(g)_ → 3Al_2_O_3_·2SiO_2(s)_ + 16HF_(g)_

In summary, the high pressure of water/fluoride vapours in the synthesis system at high temperatures could enhance the formation of crystalline mullite from AlF_3_ by accelerating the vapour phase reaction (see eqn (4)). The water vapour or air/oxygen can be supplied during the synthesis of mullite *via*: (i) continuous air/oxygen flow from outside, but this will remove some fluoride vapours also or (ii) using hydrated reactants such as AlF_3_·3H_2_O or another alumina source in a closed synthesis system. In the case of the latter approach, the evolution of gaseous oxygen or water vapours should happen around 700–800 °C, where the nucleation of mullite starts.

Here we report the fluoride-assisted synthesis of crystalline mullite from hydrous aluminium sulfate Al_2_(SO_4_)_3_·3H_2_O and AlF_3_·3H_2_O. Hydrous aluminium sulfate is chosen for the present study because it acts not only as an alumina source but also its thermal decomposition below 827 °C results in water vapour, sulfur dioxide and oxygen.^19,20^ The evolution of these gaseous species is expected to not only promote the reaction between AlOF and SiF_4_ to form mullite at low temperatures but also facilitate the growth of mullite whiskers. To confirm the role of these gaseous species in the synthesis temperature and morphology of mullite, anhydrous α-alumina and γ-alumina are also applied in the synthesis instead of hydrous aluminium sulfate. Only hydrous aluminium sulfate leads to the mullite formation at lower temperatures (800 °C) if compared with that previously reported in the literature (>1000 °C).^8,21^ The resulting mullite whiskers were in powder form, which is useful for further applications such as ceramic and metal reinforcement.

## Experimental

### Synthesis

Crystalline mullites were synthesized by the solid-state route as follows. Aluminium sulfate octadecahydrate (Al_2_(SO_4_)_3_·18H_2_O, ≥97%, Merck), α-alumina (α-Al_2_O_3_, 99.99%, AKP-50, Sumitomo) and γ-alumina (γ-Al_2_O_3_, 99.97%, Alfa Aeser) were used as an alumina source. Fumed silica (SiO_2_, 99.8%, Aerosil® OX 50, Evonik) and aluminium fluoride trihydrate (AlF_3_·3H_2_O, ≥97%, Ventron) were used as a silica source and alumina/fluorine sources, respectively. Al_2_(SO_4_)_3_·18H_2_O was first calcined separately at 300 °C for 12 hours to remove any adsorbed water molecules and to obtain stable Al_2_(SO_4_)_3_·3H_2_O. All other reagents were used without any modification. 10 mmol of alumina source (Al_2_(SO_4_)_3_·3H_2_O, α-Al_2_O_3_ or γ-Al_2_O_3_), 5 mmol AlF_3_·3H_2_O and 8.32 mmol of SiO_2_ were mixed and well-ground together in a pestle and mortar for 10 min to obtain 3Al_2_O_3_·2SiO_2_ (3 : 2 mullite). The ground powder mixtures were placed in a small alumina crucible (height = 25 mm, diameter = 20 mm) and closed with a lid. This small alumina crucible placed in another larger alumina crucible (height = 40 mm, diameter = 30 mm) and alumina paste was applied to the lid of the outer crucible to minimize escape of *in situ* formed gases, as illustrated in Fig. S1.† Then closed crucibles were heated at different synthesis temperatures (700–1000 °C) in an electric furnace (Nabertherm) for 3 hours (heating and cooling rates of 5 °C min^−1^). The furnace was equipped with an exhaust system to remove all gaseous species released during the synthesis. The naming of samples, the molar ratio of each component and reaction temperatures are presented in Table 1.

**Table 1:** Material compositions, synthesis conditions and summarized characteristics of products obtained with different conditions

	*m*(aluminium source), g		*m*(SiO_2_), g	*m*(AlF_3_·3H_2_O), g	Synthesis temperature, °C	Phase composition	Crystal shape	Average length and diameter of whiskers, μm
A1	Al_2_(SO_4_)_3_·3H_2_O	3.96	0.5	0.69	700	Aluminum sulfate and aluminum fluoride	Irregular	—
A2	Al_2_(SO_4_)_3_·3H_2_O	3.96	0.5	0.69	800	Mullite	Needle-like particles	0.59 ± 0.2 and 0.22 ± 0.06
A3	Al_2_(SO_4_)_3_·3H_2_O	3.96	0.5	0.69	900	Mullite	Needle-like particles	2.02 ± 0.30 and 0.15 ± 0.05
A4	Al_2_(SO_4_)_3_·3H_2_O	3.96	0.5	0.69	1000	Mullite	Needle-like particles	2.37 ± 1.70 and 0.18 ± 0.11
A5	α-Al_2_O_3_	1.02	0.5	0.69	1000	Corundum, cristobalite and topaz	Small round, large round, and bar-like particles	—
A6	γ-Al_2_O_3_	1.02	0.5	0.69	1000	Amorphous silica, δ-alumina and mullite	Irregular	—

### Characterization

Crystalline phases in the final products were identified by powder X-ray diffraction (XRD, D8 Advance, Brucker, Germany) equipped with a Lynx Eye 1D detector and using Co Kα radiation. The diffraction patterns were collected in a Bragg–Brentano geometry. Rietveld refinement was performed using the FULLPROF program.^22^

The morphology and elemental compositions of the samples were examined by scanning electron microscopy (SEM) in LEO Gemini 1530, Carl (Zeiss, Germany) coupled with an energy dispersive X-ray detector (Thermo Fisher Scientific, USA) on samples sputtered with a gold layer. Transmission electron microscopy (TEM) images of mullite whiskers were obtained on an FEI Tecnai G2 20 S-TWIN electron microscope equipped with an energy dispersive X-ray detector (EDX) operated at 200 kV (FEI, USA).

To address the formation mechanisms of mullite, thermogravimetric (TG) and differential thermal analysis (DTA) were performed under a mixture atmosphere of oxygen and argon atmosphere (20% O_2_ – 80% Ar) using STA 449F3 (Netzsch, Germany).

## Results and discussion

**Fig. 1:**
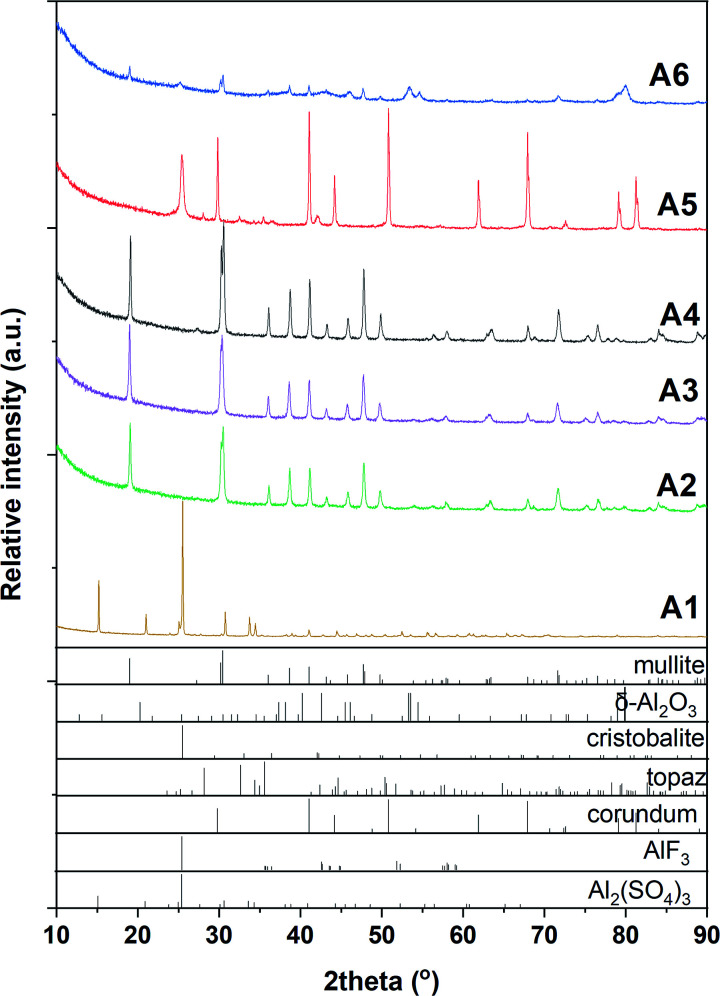
XRD patterns of samples synthesized using Al_2_(SO_4_)_3_·3H_2_O at 700 °C (A1), 800 °C (A2), 900 °C (A3) and 1000 °C (A4). α-Al_2_O_3_ (A5) and γ-Al_2_O_3_ (A6), both – synthesized at 1000 °C.

According to XRD results (Fig. 1 and Table 1), phase-pure mullite was obtained from the fluoride-assisted reaction between hydrated aluminium sulfate, AlF_3_ and SiO_2_ at *T* ≥ 800 °C for 3 hours. In contrast, no mullite is formed at a lower temperature (700 °C) and crystalline aluminium sulfate and aluminium fluoride are still present in the sample in addition to amorphous silica.

**Fig. 2:**
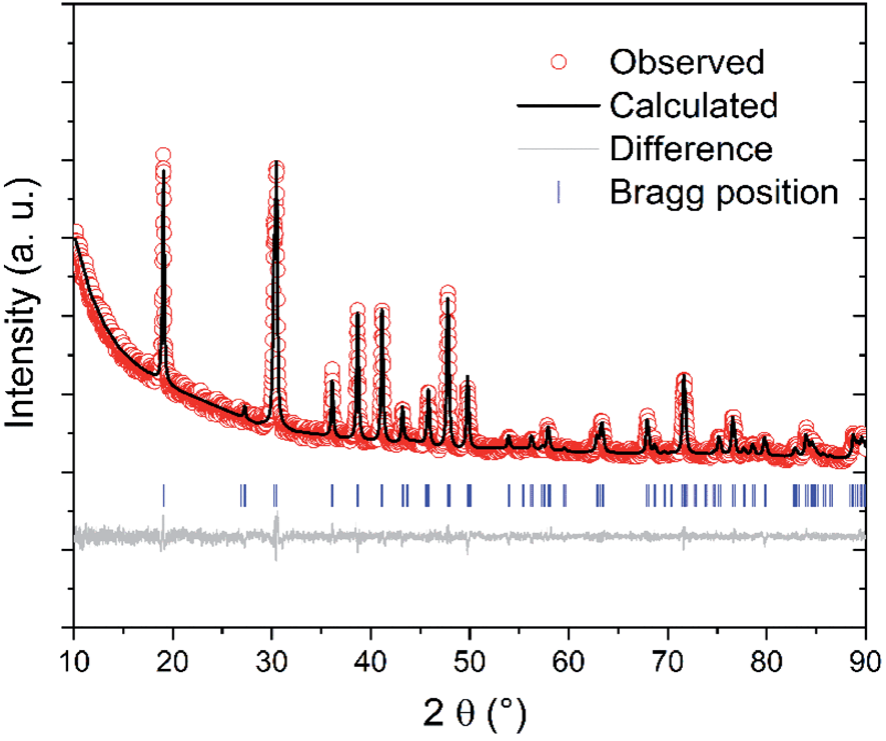
Rietveld refinement of X-ray powder diffraction data for A2 sample showing the observed (red circle) and calculated (solid black line) intensities, the calculated Bragg reflections (blue tick marks), and the difference (solid grey line).

Rietveld refinement of XRD data (Fig. 2 and Table 2) confirm the crystal structure of mullite (space group *Pbam*, no. 55) and reveals that the lattice parameters *a* and *b* of the mullite synthesized at 1000 °C are lower than those of the mullites synthesized at 800–900 °C. In contrast, no remarkable change in the lattice parameter *c* is observed with increasing the synthesis temperature. These results are in good agreement with previous studies reporting similar changes in the lattice parameters of mullite with synthesis temperatures due to the change of structural order of the mullite lattice with temperature.^23^ The mullite becomes more crystallized and ordered with increasing the synthesis temperature. This finding is also confirmed by the bigger crystallite size and low microstrain of the mullite synthesized at 1000 °C if compared with those synthesized at lower temperatures (800–900 °C). Moreover, Rietveld refinement reveals that all the samples have preferred orientation along the *c* axis (the [001] direction). This anisotropic growth of mullite crystals might be due to the fact that mullite whiskers are usually formed without constraints during vapour–solid reaction.^28^ Moreover, the activation energy for grain growth along *c* axis is lower than along *a* and *b* axes.^29^

**Table 2:** Rietveld refinement results for the samples obtained at different temperatures

Samples	Synthesis temperature (°C)	Lattice parameters (Å)	Unit cell volume (Å^3^)	Crystallite size (nm)	Microstrain ×10^−4^
A2	800	*a* = 7.5957(3)	168.87(1)	49.8(6)	1.28
		*b* = 7.6980(3)			
		*c* = 2.8881(1)			
A3	900	*a* = 7.6029(4)	168.94(2)	49.9(7)	1.35
		*b* = 7.6922(4)			
		*c* = 2.8886(2)			
A4	1000	*a* = 7.5625(3)	168.05(1)	75.7(8)	1.10
		*b* = 7.6897(3)			
		*c* = 2.8898(1)			

This work shows that crystalline mullite can be synthesized by fluoride-assisted route as low as 800 °C using aluminium sulfate as an alumina source *via* fluoride-assisted reaction. This synthesis temperature is much lower than that reported (≥1000 °C) when alumina, kaolin or topaz are used in the synthesis of mullite whiskers.^10,21^ This finding was also confirmed in this work. As shown in (Fig. 1a), no crystalline mullite phase was formed in the sample A5 even at 1000 °C when α-Al_2_O_3_ is used instead of hydrated aluminium sulfate. Only amorphous silica phase is crystalized into cristobalite phase and a small amount of topaz (Al_2_SiO_4_(F, OH)_2_) is formed. For the sample A6, a very small amount of mullite was observed in addition to the reactant phases. Interestingly, no topaz or cristobalite phases are detected in this sample. As has been reported, topaz could be formed from the reaction of AlF_3_ with SiO_2_ at 700 °C, before decomposing into mullite phase above 1000 °C.^24^ However, the transition temperature of topaz into mullite depends on the pressure of SiF_4_ in the synthesis system.^25^ This could be the reason for the formation of a small amount of mullite in the sample A6. The different reactivity of various alumina phases could be another reason for the formation of mullite in sample A6 but not in sample A5; similar results were reported with α- and κ-alumina phases.^26^

**Fig. 3:**
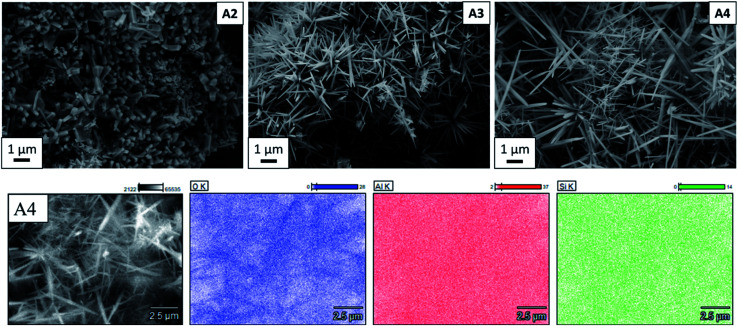
At the top: SEM images of mullite whiskers synthesized from aluminium sulfate at 800 °C (A2), 900 °C (A3) and 1000 °C (A4). At the bottom: EDX analysis of A4 sample.

As can be seen in Fig. 3, A2, A3 and A4 samples synthesized using Al_2_(SO_4_)_3_·3H_2_O as alumina source at 800 °C, 900 °C, and 1000 °C, respectively, are composed of needle-like mullite crystals. EDX mapping (Fig. 3) also shows the uniform and equal distribution of Al and Si elements, which indicates mullite formation without other phases. As listed in Table 1, the average length of whiskers, measured by the image analyzing software ImageJ,^27^ increased from 0.59 ± 0.2 μm to 2.37 ± 1.7 μm with increasing synthesis temperature from 800 °C to 1000 °C. In contrast, no significant change in the average diameter of whiskers is observed with increasing the synthesis temperature. The morphology of mullite synthesized in this work is consistent with previous works showing the formation of mullite whiskers *via* vapour-phase reaction of xerogel, derived from tetraethoxysilane and aluminium nitrate nonahydrate, with AlF_3_ at 1200 °C in a closed alumina crucible.^8^ Rashad *et al.* also reported similar microstructure with the reaction of kaolin clay and AlF_3_ at 1300 °C in a closed crucible.^12^ Sample A5 synthesized from α-Al_2_O_3_ contains particles with different morphologies (Fig. S2†). The results of EDX mapping (Fig. S3†) show that elemental composition of the large round particles are Si and O, whereas the small round particles are Al and O. This reveals that the large round particles (glass-like) are cristobalite, the small round particles are unreacted alumina (corundum), and then the very small amount of bar-like particles are topaz. Irregular particle shapes of amorphous silica and alumina polymorphs are observed in samples A1 and A6 (Fig. S2†) synthesized from Al_2_(SO_4_)_3_·3H_2_O at 700 °C and γ-Al_2_O_3_ at 1000 °C, respectively. These bar-like or needle-like microstructures are characteristic for topaz and mullite obtained by fluoride-assisted reactions.^8,11,21,26^

**Fig. 4:**
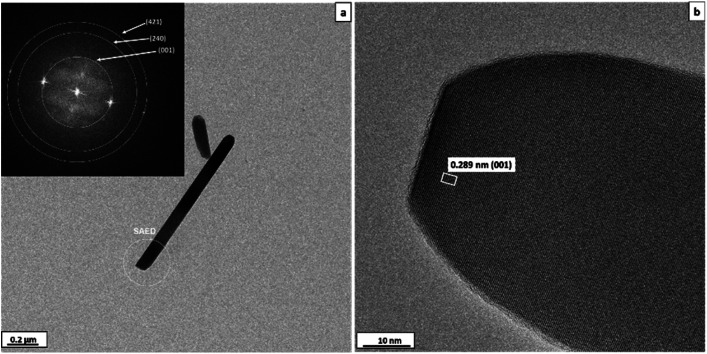
TEM image of mullite whiskers synthesized from aluminium sulfate at 1000 °C. (a) Morphology of whisker, SAED pattern inset, (b) high resolution image. Inset in (a) shows the fast Fourier transform (FFT) pattern.

The morphology and crystal structure of mullite whiskers formed at 1000 °C are also confirmed by HRTEM analysis (Fig. 4). The fast Fourier transform (FFT) pattern (inset of Fig. 4a) reveals the diffraction rings corresponding to the (001), (240) and (421) planes with *d* spacings of 0.289, 0.172 and 0.146 nm, respectively, of mullite (space group *Pbam*, no. 55). Average fringe distances of 0.289 nm corresponding to the (001) plane distance is also observed in the HRTEM image of the sample (Fig. 4b). These results are in good agreement with the XRD results.

**Fig. 5:**
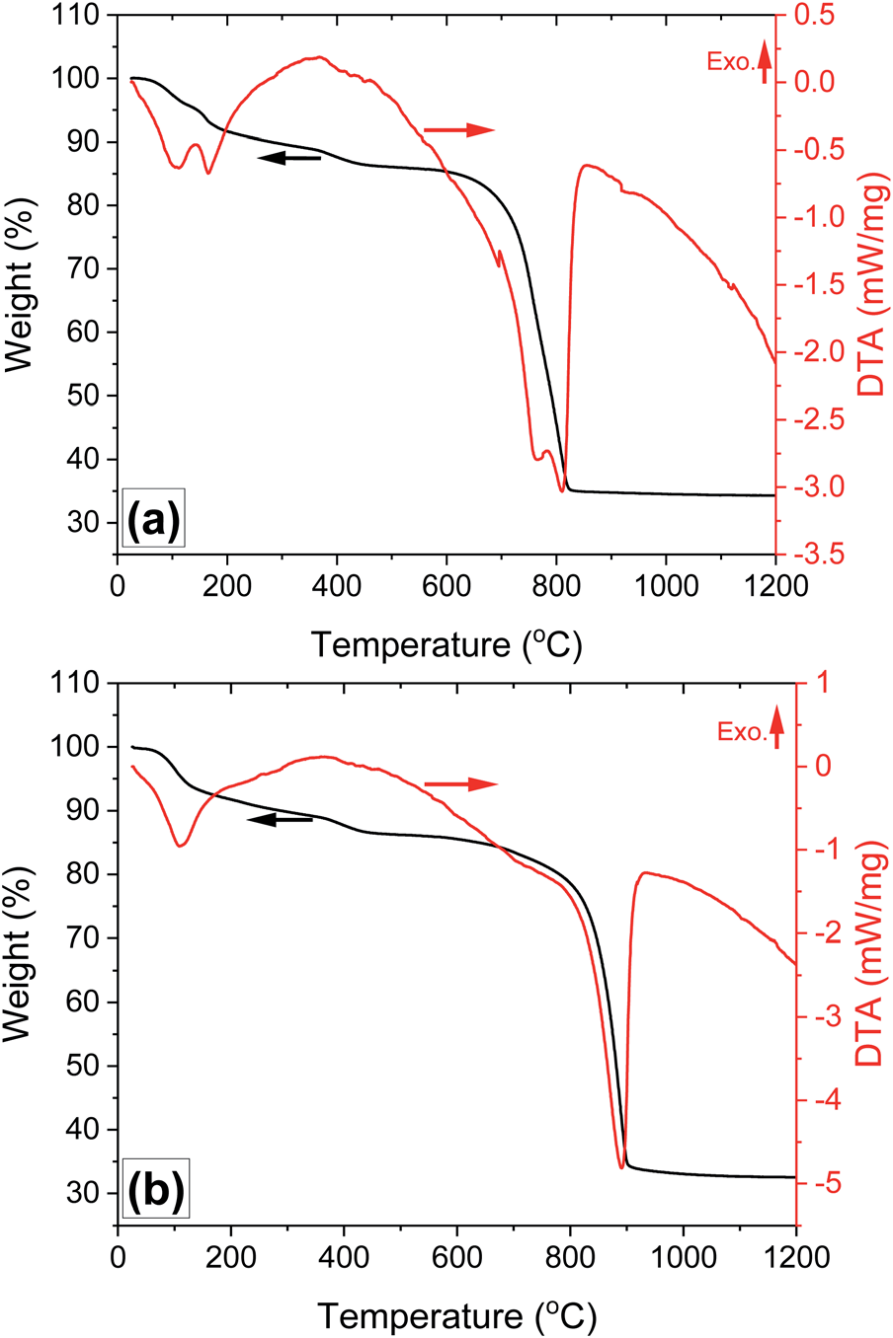
Results of the TGA-DTA characterization of the (a) Al_2_(SO_4_)_3_·3H_2_O + SiO_2_ + AlF_3_·3H_2_O; and (b) Al_2_(SO_4_)_3_·3H_2_O + SiO_2_ powder mixture without AlF_3_·3H_2_O.

The two endothermic peaks (Fig. 5a) observed below 200 °C and accompanied with a weight loss of 9 wt%, can be attributed to the evaporation of absorbed moisture and evaporation of chemically bonded water from AlF_3_·3H_2_O, which is in agreement with the literature.^30^ This finding is also confirmed from the TG-DTA curve measured during the thermal decomposition of AlF_3_·3H_2_O (Fig. S4a†). The main decomposition stage is observed in the temperature range 660–820 °C, accompanied by two strong endothermic peaks and weight loss of 49 wt%. No further weight loss is observed above 825 °C, suggesting that the decomposition process is complete. As has been reported, Al_2_(SO_4_)_3_·3H_2_O first undergoes partial dehydration below 600 °C, and the remaining water evaporation occurs together with the decomposition of Al_2_(SO_4_)_3_ to Al_2_O_3_ and evolution of gaseous SO_2_ and O_2_ at higher temperatures (>850 °C).^19,20^ In this work, the partial dehydration of Al_2_(SO_4_)_3_·3H_2_O occurs between 330 °C and 560 °C with weight loss of 4 wt% (Fig. S4b†), and the latter decomposition stage of Al_2_(SO_4_)_3_ to Al_2_O_3_ is shifted to a much lower temperature (660–820 °C) in the presence of AlF_3_ (Fig. 5a and S4c†). In contrast, no remarkable change in the thermal decomposition behaviour of Al_2_(SO_4_)_3_ into Al_2_O_3_ is observed in the presence of only SiO_2_ and absence of AlF_3_ (Fig. 5b). The lower decomposition temperature of Al_2_(SO_4_)_3_ to Al_2_O_3_, O_2_ and SO_2_ in the presence of AlF_3_ can be explained by the possible chemical reactions between produced gaseous oxygen with AlF_3_ (see reactions (1) and (4)). This finding is consistent with previous work showed that a small concentration of H_2_ in argon atmosphere could shift the decomposition of Al_2_(SO_4_)_3_ to lower temperature, in comparison with air atmosphere, due to the reaction between H_2_ and the produced O_2_.^31^ Moreover, only one exothermic peak is observed during thermal decomposition of Al_2_(SO_4_)_3_·3H_2_O (Fig. S4a†), Al_2_(SO_4_)_3_·3H_2_O + SiO_2_ (Fig. 5b), Al_2_(SO_4_)_3_·3H_2_O + AlF_3_·3H_2_O (Fig. S4c†) at temperatures above 800 °C, whereas no chemical reaction takes place. In contrast, two endothermic peaks occur during the formation of the A4 sample from its corresponding composition at 770 °C and 810 °C. The first endothermic peak might be due to the decomposition of Al_2_(SO_4_)_3_ to Al_2_O_3_ and gaseous products. The second endothermic peak observed at 810 °C in the DTA spectra of the A4 might be attributed to the formation of mullite. These results are consistent with previously reported works showed that the formation of mullite is an endothermic process.^32^ The overlapping of the endothermic peaks related to the formation of Al_2_O_3_ from Al_2_(SO_4_)_3_ with that attributed to the formation of mullite suggests that the mullite phase is formed *in situ* during the decomposition of Al_2_(SO_4_)_3_.

The *in situ* formation of mullite is also confirmed by quenching experiments on heated powder mixtures of Al_2_(SO_4_)_3_·3H_2_O (2 mmol) and AlF_3_·3H_2_O (1 mmol) in the absence and presence of SiO_2_ (1.66 mmol) at 825 °C. XRD characterization of the quenched samples reveals that a small amount of corundum (α-Al_2_O_3_) is formed in the absence of SiO_2_ (Fig. S5a†); otherwise, Al_2_O_3_ reacts with amorphous silica to form mullite (Fig. S6b†).

According to the XRD and TG-DTA results, the low-temperature *in situ* formation (800 °C) of mullite from Al_2_(SO_4_)_3_·3H_2_O and SiO_2_ in the presence of AlF_3_·3H_2_O can be explained by: (i) the high chemical reactivity of Al_2_O_3_ phase resulted from the thermal decomposition of Al_2_(SO_4_)_3_·3H_2_O, (ii) the high pressure of fluoride/water vapours inside the reaction system, which drives them easily to supersaturation within the crucibles. This highly reactive Al_2_O_3_ reacts first with fluorine gas to form AlOF (eqn (2)) and further on with SiF_4_ in the presence of H_2_O vapour to form mullite (eqn (4)).

## Conclusions

Crystalline mullite whiskers have been synthesized by fluoride-assisted synthesis from aluminium sulfate, aluminium fluoride and fumed silica at 800–1000 °C. The crystal structure of the synthesized mullite has been confirmed by Rietveld refinement of the powder XRD data. This work showed that aluminium sulfate could be a promising starting material to obtain mullite at low temperatures in the presence of a small amount AlF_3_, rather than costly precursors and complex synthesis methods used in literature. Additionally, the obtained mullite whiskers were in powder form, where commercially available mullite powders are obtained by crushing solid sintered bodies, which requires extra processing cost. Mullite whiskers have great potential for the reinforcement of ceramics and metals. A further study is needed to explore the detailed mechanism of whisker formation in the systems where both fluorine and oxygen are present.

## Conflicts of interest

There are no conflicts to declare.

## Supplementary Material

RA-010-D0RA05997H-s001
