# Supplementary material for: Low-temperature fluoride-assisted synthesis of mullite whiskers
Source: RSC Adv. 2020 Aug 24;10(52):31180–6. doi: 10.1039/d0ra05997h (PMC9056419; doi:10.1039/d0ra05997h)
Supplement: RA-010-D0RA05997H-s001 [file RA-010-D0RA05997H-s001.pdf]

Electronic Supplementary Material (ESI) for RSC Advances.
This journal is © The Royal Society of Chemistry 2020
Please do not adjust margins

Supplementary Information

Low-Temperature Fluoride-assisted Synthesis of Mullite Whiskers

Amanmyrat Abdullayev, Fabian Zemke, Aleksander Gurlo and Maged F. Bekheet*

Fachgebiet Keramische Werkstoffe / Chair of Advanced Ceramic Materials, Institute of Materials Science and Technology, Technische Universität Berlin, 10623 Berlin, Germany

Email: maged.bekheet@ceramics.tu-berlin.de

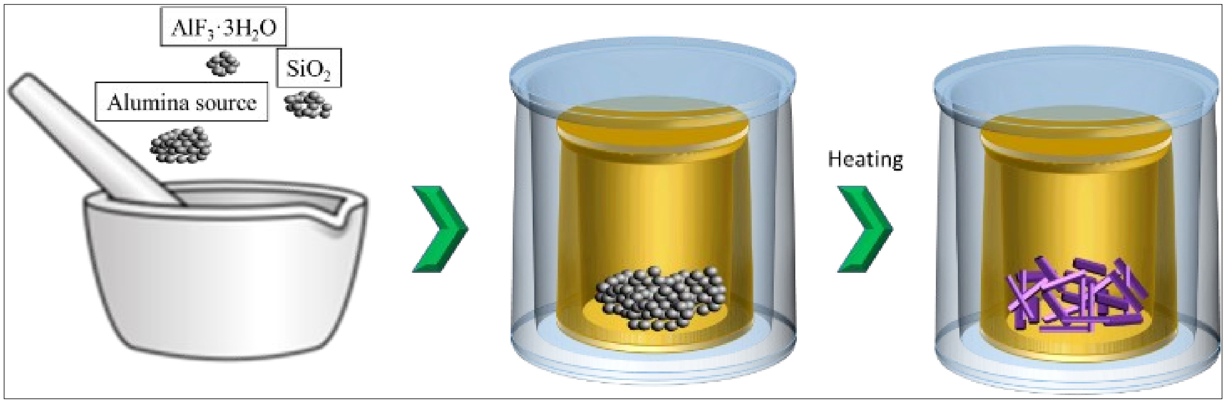

Figure S1. Illustration of mullite preparation, where after manual mixing, powders placed in a small crucible and closed. This closed small crucible placed in a larger crucible and closed to prevent the escape of reactive gases from the system, then heated to synthesize mullite.

Please do not adjust margins

Please do not adjust margins

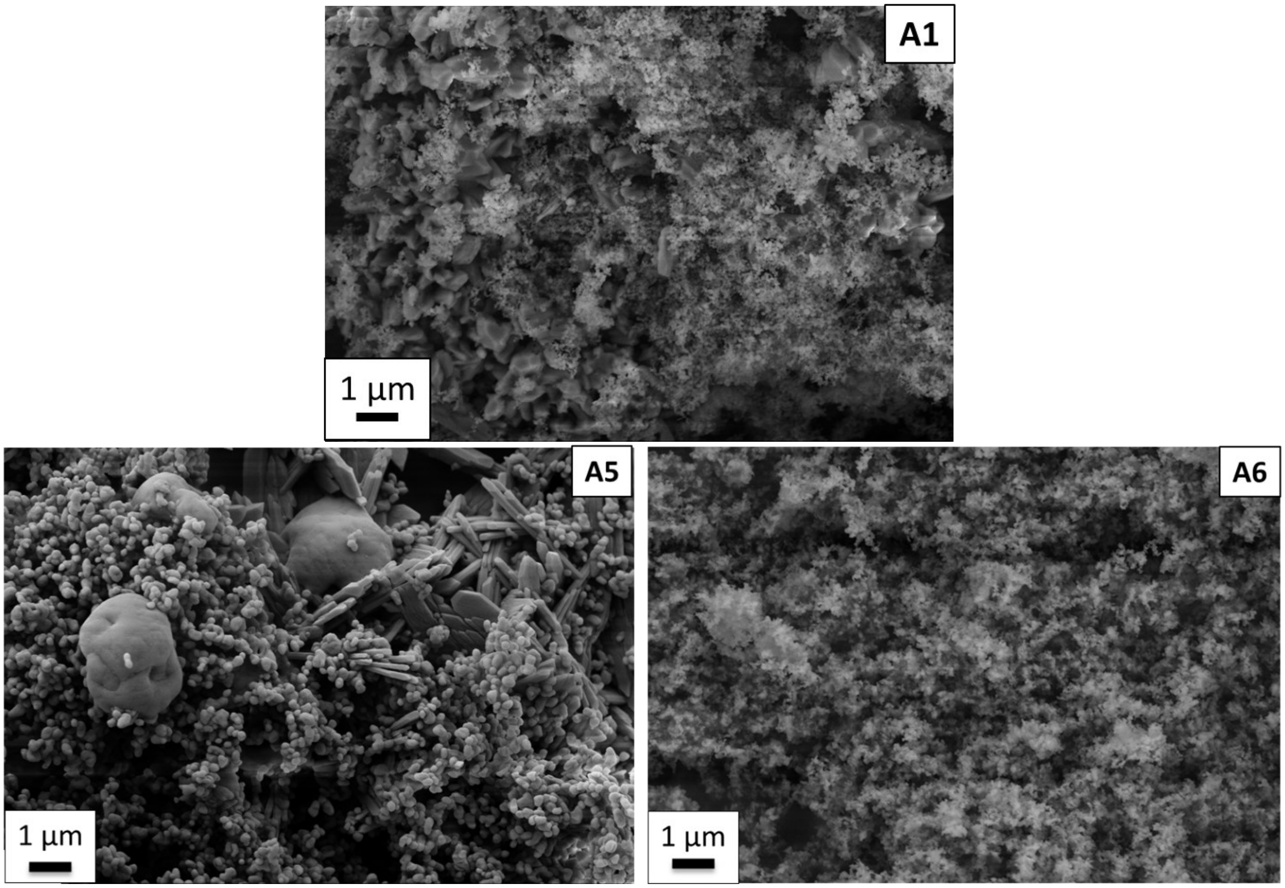

Figure S2. SEM images of A1, A5 and A6. A1, an alumina source is aluminium sulphate and synthesis temperature is 700°C, show irregular particle morphology. A5, an alumina source is α-alumina, exhibited very small round unreacted alumina, large round cristobalite and small amount bar-like topaz particles. A6, an alumina source is γ-alumina, exhibited irregular particles of amorphous silica and polymorph of alumina.

Please do not adjust margins

Please do not adjust margins

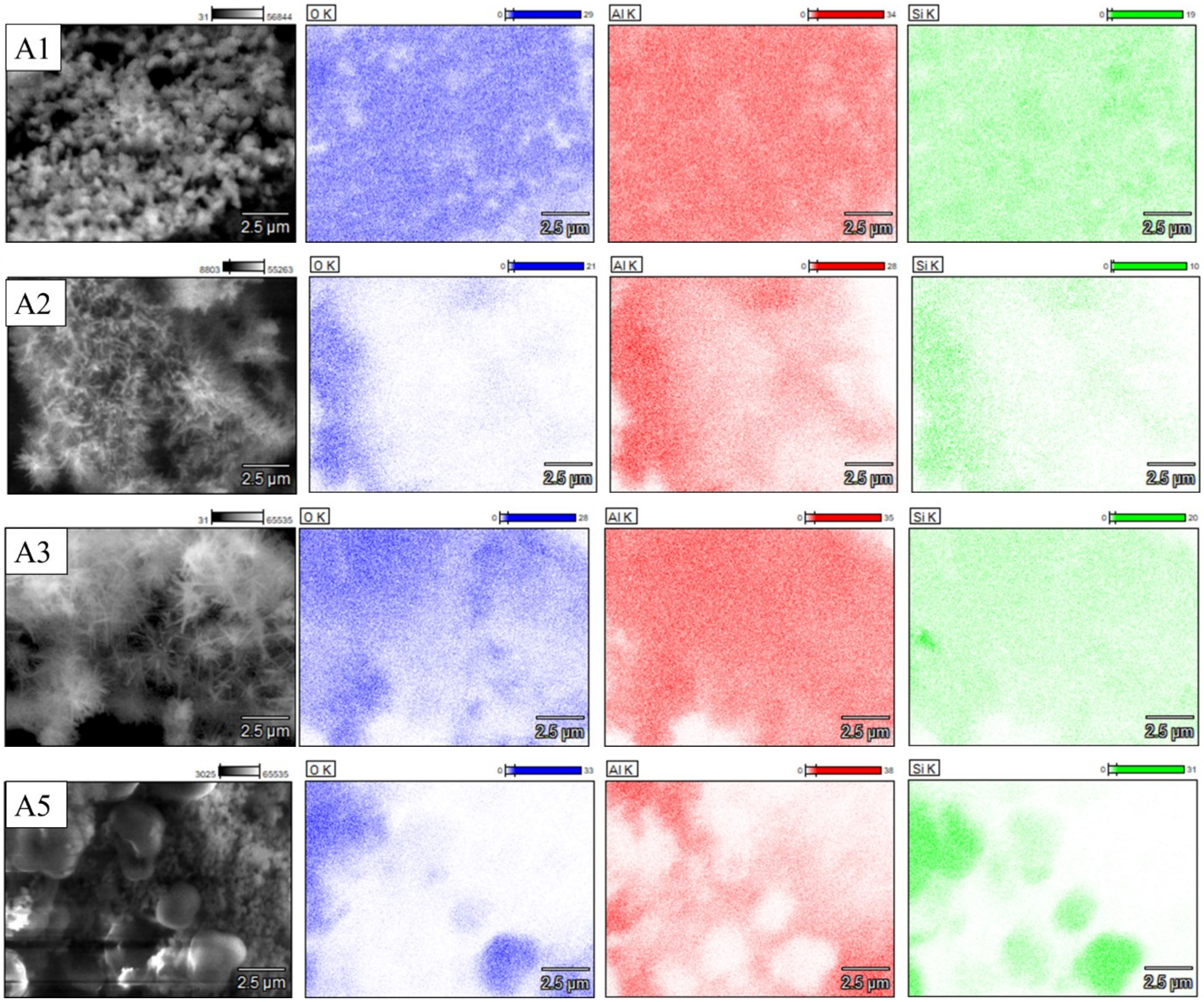

Figure S3. SEM and EDX mapping of A1 to A5. Samples usually presented evenly distributed Al, Si and O, except A5, which clearly indicates small round particles are unreacted alumina and large round particles are crystallized silica.

This journal is © The Royal Society of Chemistry 20xx

Please do not adjust margins

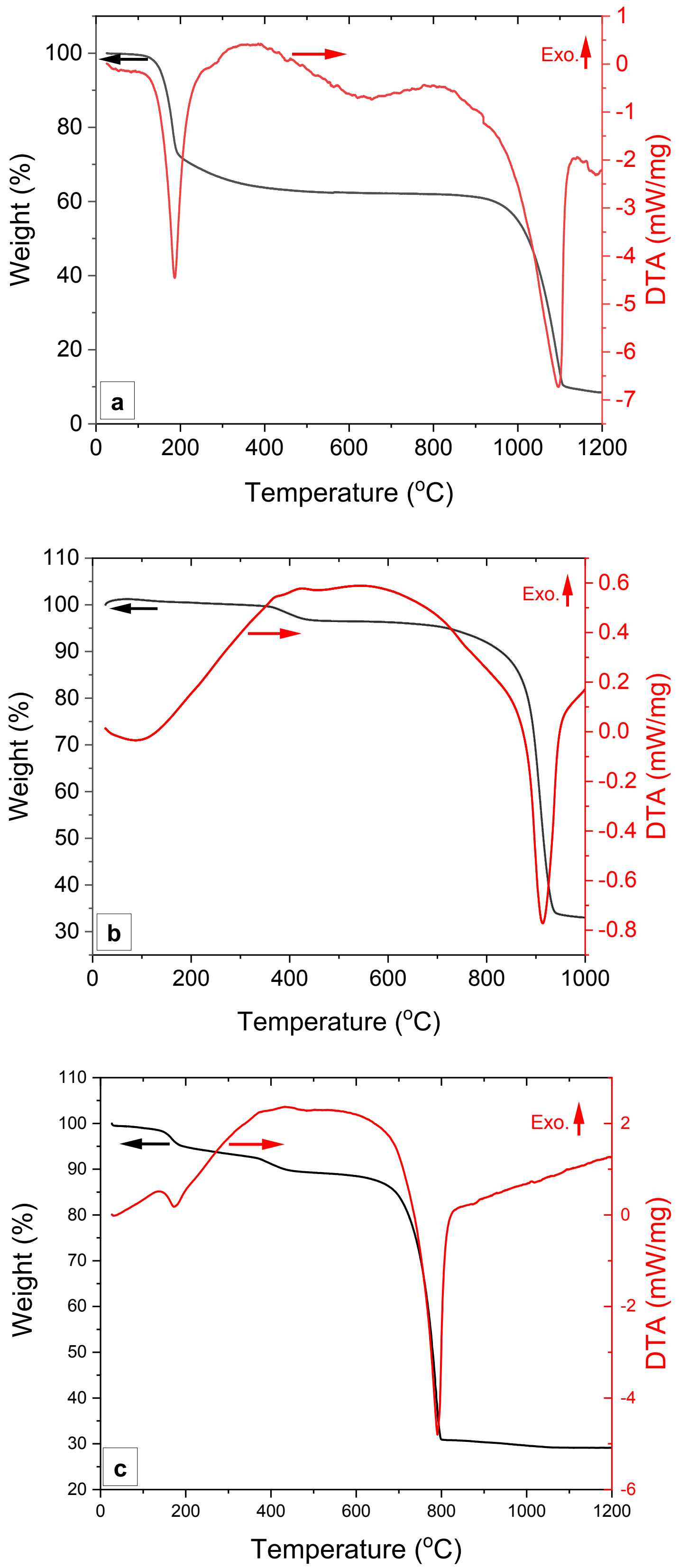

Figure S4. TGA and DTA results of a) $AlF_3 \cdot 3H_2O$; b) $Al_2(SO_4)_3 \cdot 3H_2O$; c) mixture of $Al_2(SO_4)_3 \cdot 3H_2O$ + $AlF_3 \cdot 3H_2O$.

This journal is © The Royal Society of Chemistry 20xx

Please do not adjust margins

Please do not adjust margins

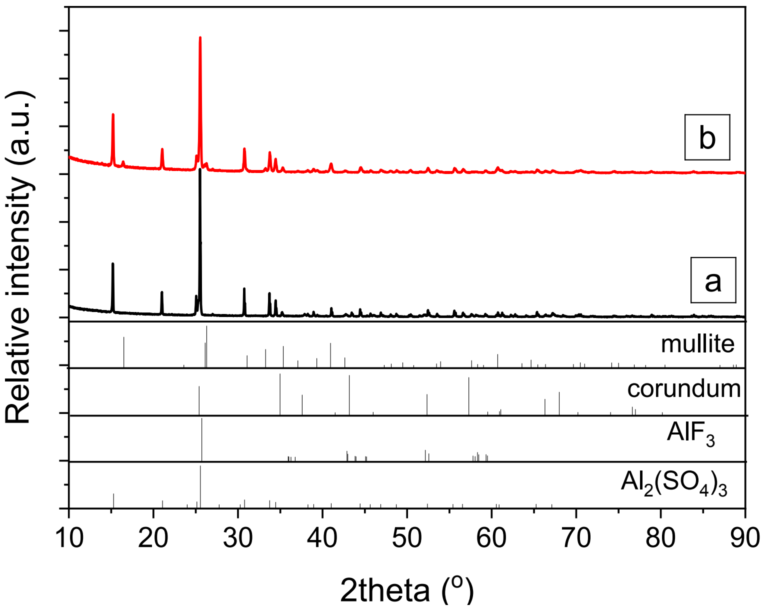

Figure S5. XRD patterns of quenching experiments, where mixture of $Al_2(SO_4)_3{\cdot}3H_2O$ + $AlF_3{\cdot}3H_2O$ with molar ratio of 2:1 (a) and mixture of $Al_2(SO_4)_3{\cdot}3H_2O$ + $AlF_3{\cdot}3H_2O$ + SiO_2 with a molar ratio of 2:1:1.66 (b) heated until 825 ^oC in a closed alumina crucible and then quenched with liquid nitrogen.

This journal is © The Royal Society of Chemistry 20xx

Please do not adjust margins
